# Light-Based Anti-Biofilm and Antibacterial Strategies

**DOI:** 10.3390/pharmaceutics15082106

**Published:** 2023-08-09

**Authors:** Ambreen Kauser, Emilio Parisini, Giulia Suarato, Rossella Castagna

**Affiliations:** 1 Department of Biotechnology, Latvian Institute of Organic Synthesis, Aizkraukles 21, LV-1006 Riga, Latvia; ambreen.kauser@osi.lv (A.K.); emilio.parisini@osi.lv (E.P.); 2 Faculty of Materials Science and Applied Chemistry, Riga Technical University, Paula Valdena 3, LV-1048 Riga, Latvia; 3 Department of Chemistry “G. Ciamician”, University of Bologna, Via Selmi 2, 40126 Bologna, Italy; 4 Istituto di Elettronica e di Ingegneria dell’Informazione e delle Telecomunicazioni, Consiglio Nazionale delle Ricerche, CNR-IEIIT, Piazza Leonardo da Vinci 32, 20133 Milano, Italy; 5 Dipartimento di Chimica, Materiali e Ingegneria Chimica “G. Natta”, Politecnico di Milano, Piazza Leonardo da Vinci 32, 20133 Milano, Italy

**Keywords:** antimicrobial resistance, biofilm, EPS, AHLs, AIPs, quorum sensing inhibition, bacterial adhesion, photopharmacology, hydrogels, nanoparticles, light-triggered drug delivery

## Abstract

Biofilm formation and antimicrobial resistance pose significant challenges not only in clinical settings (i.e., implant-associated infections, endocarditis, and urinary tract infections) but also in industrial settings and in the environment, where the spreading of antibiotic-resistant bacteria is on the rise. Indeed, developing effective strategies to prevent biofilm formation and treat infections will be one of the major global challenges in the next few years. As traditional pharmacological treatments are becoming inadequate to curb this problem, a constant commitment to the exploration of novel therapeutic strategies is necessary. Light-triggered therapies have emerged as promising alternatives to traditional approaches due to their non-invasive nature, precise spatial and temporal control, and potential multifunctional properties. Here, we provide a comprehensive overview of the different biofilm formation stages and the molecular mechanism of biofilm disruption, with a major focus on the quorum sensing machinery. Moreover, we highlight the principal guidelines for the development of light-responsive materials and photosensitive compounds. The synergistic effects of combining light-triggered therapies with conventional treatments are also discussed. Through elegant molecular and material design solutions, remarkable results have been achieved in the fight against biofilm formation and antibacterial resistance. However, further research and development in this field are essential to optimize therapeutic strategies and translate them into clinical and industrial applications, ultimately addressing the global challenges posed by biofilm and antimicrobial resistance.

## 1. Introduction

Antimicrobial resistance (AMR) occurs when microbes (bacteria, viruses, fungi, and parasites) change over time and no longer respond to the action of drugs (antibiotics, antivirals, antifungals, and antiparasitics) that would normally kill them or limit their growth. In this regard, bacterial resistance to antibiotics is of paramount importance in the One Health context. Bacteria can indeed feature AMR, a process that is enabled by different mechanisms and can be classified into (I) intrinsic, (II) acquired, and (III) adaptive. While intrinsic AMR relates to all those inherent mechanisms that are adopted by bacteria to inhibit antimicrobial agents and that do not require any contact with the antimicrobial agents themselves, acquired resistance is the result of either an exchange of genetic material or mutations. This type of resistance is more common in biofilms due to cell proximity [1]. Moreover, altered gene expression can result from exposure to environmental stress [2]. It has also been suggested that adaptive resistance may arise from a change in the intrinsic resistance mechanisms, leading to an increase in AMR in response to environmental stress and mutations [3].

Antibiotics, which have been widely used for more than half a century to treat bacterial infections, have greatly contributed to the promotion of human health and life expectancy [4,5]. This significant impact of antibiotics has been undermined in recent years due to growing levels of antimicrobial resistance [6]. Persistent consumption of antibiotics, genetic variations, and exposure to infections in hospitals can favor the selection and spread of multidrug-resistant bacteria, which has enormous implications for worldwide healthcare delivery and population health [7,8]. According to the antimicrobial resistance report published in 2016, the number of deaths caused by pathogenic bacteria is anticipated to rise to 10 million by 2050 if no urgent action is taken to reverse this course [9]. To this end, many approaches are being explored by the scientific community, with variable success.

In this review, we describe light-triggered approaches that target different stages of biofilm formation and maturation. These include surface patterning, pharmacological interventions, and the use of smart materials. Moreover, we focus on those light-responsive materials and photo-cleavable or photoswitchable molecules that have been designed for antibacterial applications. In the last decades, the photopharmacology approach has been successfully applied to different biological targets, leading to a series of molecular tools for the functional modulation of ion channels [10,11,12], glutamate receptors [13], G-protein-coupled receptors [14,15,16], protein-protein interactions [17,18], and enzymes [19], all of which have been successfully tested in vitro and in vivo for different therapeutic applications [20,21,22,23].

The use of light as a trigger appears to be particularly promising, as several classes of organic and inorganic materials can respond to a broad spectrum of wavelengths [24,25,26,27,28,29]. Moreover, certain wavelengths of light are not harmful for humans or the environment. This approach allows a non-invasive, on/off regulation of the material properties and activity, ultimately leading to enhanced control of the irradiation site and dosage [26].

## 2. Biofilm Formation and Development

**Figure 1:**
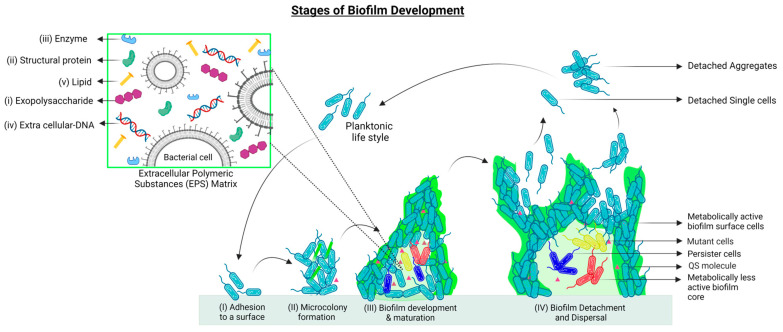
Stages of biofilm development: (I) Attachment of free-living bacteria to a compatible surface using physical forces, cell appendages, and secreted adhesins. (II) Microcolony formation to maintain surface attachment is accompanied by the initial production of Extracellular Polymeric Substances (EPS, in light green). (III) Biofilm maturation is achieved through the establishment of a microenvironment suitable for cellular heterogeneity (depicted as red, blue, and yellow cells), the release of quorum sensing molecules (pink triangles), and enhanced EPS matrix production to combat environmental stress. (IV) At the time of nutrient depletion and accumulation of toxic compounds, dispersal of biofilm occurs. Cells can detach individually and in aggregates to start either planktonic life or grow into new biofilms. Zoomed section. Composition of the EPS matrix: The EPS matrix contains an array of biofunctional molecules such as (i) exopolysaccharides, essential for surface adhesion and structural integrity of biofilms; (ii) structural proteins, which connect the cells to the EPS and stabilize the biofilm architecture; and (iii) extracellular enzymes (mostly hydrolases and lyases) that facilitate the degradation of EPS molecules into simpler products to be used by the biofilms as sources of energy and carbon. The EPS degradation process is also important for biofilm dispersal and the formation of new biofilms. (iv) eDNA acts as an intercellular connector in the matrix and as a facilitator of Horizontal Gene Transfer (HGT). (v) Lipids are essential for bacterial adhesion to hydrophobic surfaces. The figure is partially adapted from [49].

To survive harsh environmental conditions, some bacterial species can live in close proximity and form highly structured multicelluar communities called biofilms, which attach to surfaces and interfaces. Inside biofilms, bacterial cells show features that are distinct from those that they show in their planktonic state, such as reduced motility and metabolic activities, heterogeneity of gene expression, inter-communal division of labor, and enhanced tolerance to antibiotics [30] (Figure 1).

In the 1670s, biofilms as complicated bacterial structures were first recognized by the Dutch microscopist Anton Van Leeuwenhoek when working on dental plaques. With the advent of electron microscopy, it was later revealed that to anchor themselves at the infection site, bacterial communities form biofilms, in which the bacteria are embedded in self-secreted extracellular viscous polymeric substances (EPSs). In most cases, the biofilm matrix accounts for around 90% of the total biofilm mass, and it is composed of exopolysaccharides, amyloid-like proteins, lipids, and extracellular DNA (eDNA) [31,32,33]. Most exopolysaccharides are species-dependent and contain repeated sugar units of the same and different types that are responsible for their polycationic or polyanionic nature [34]. Those charged molecules are essential for water retention in the biofilms and to hydrate the environment, a feature required to protect the bacterial cells in the biofilm from desiccation due to water stress, hence keeping the non-rigid structure of the biofilm with different viscosities to allow cell movements in the matrix [35]. These properties of the EPS matrix provide mechanical support to protect the resident cells from external forces, such as fluid shear, and to ensure that the biofilm community remains attached to a surface. In the context of infectious biofilms, it is difficult for neutrophils to access biofilm-forming bacterial cells because, during phagocytosis, they can only exert stress up to 1 kPa, which is not enough to break the biofilm into small pieces. Moreover, neutrophils can only ingest pathogens smaller than 10 μm; therefore, living in clusters within biofilms eventually protects bacteria from being attacked [36].

Biofilms represent the main cause of infections, such as catheter-associated urinary tract infections. Unlike planktonic cells, the bacterial population in the biofilms exerts their action by reducing their motility and metabolic activities while, at the same time, upregulating their production of extracellular toxins at the site of infection, which ensures the maximum possible tissue damage. The overall result is an abundant release of nutrient supplies, leading to further cementation of biofilm [37]. At the same time, adjacent tissues are also colonized by the shedding of daughter planktonic cells. Eventually, this spread of infection and development of new biofilms would mature into a densely packed structure that is difficult to eradicate, hence triggering chronic and recurrent infections [38,39,40].

Bacteria switch from planktonic life to biofilm mode by utilizing a combination of van der Waals, electrostatic, and hydrophobic interactions to attach themselves reversibly to biotic or abiotic surfaces through fimbriae, pili, flagella, and glycocalyx. These attachments are easily affected by the substratum type, the hydrodynamics, and other characteristics of the aqueous medium. At some point, bacteria either commit themselves to the biofilm irreversibly or revert back to their planktonic lifestyle [41,42].

In cases of conducive conditions for growth and differentiation, a biofilm develops into spatially arranged 3D structures, interspersed with fluid-filled channels, where nutrients, oxygen, and essential substances can diffuse and circulate in each individual microenvironment for the embedded microbial cells to undergo coordinated community growth that leads to the formation of microcolonies [43]. In this way, bacteria display coordinated group behavior (secretion of virulence factors, formation of biofilm), which is based on a density-dependent signal called quorum sensing [44,45]. With the formation of biofilms, bacterial cells now have distinct features as compared to their planktonic lifestyle, such as the presence of an EPS matrix, increased nutrient supply, upregulated synthesis and secretion of extracellular material, and chemical and/or electrical interactions [46]. However, triggered by various environmental factors, the biofilm can lose its stability, either actively or passively, thus dispersing into its surroundings via the detachment of either single cells or large aggregates of cells [35,47,48], which can then land at new locations to initiate the formation of a new colony [49].

### Quorum Sensing

**Figure 2:**
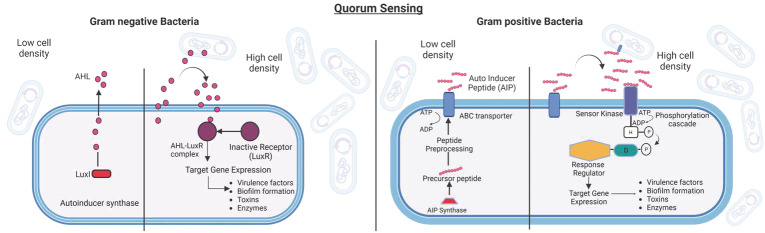
(**Left**): Quorum Sensing (QS) in Gram-negative Bacteria. Gram-negative bacteria follow the LuxL-LuxR QS regulatory system. The autoinducer synthase (LuxI) secretes QS signaling molecules, AHLs (pink circles), that, upon reaching the threshold concentrations, enter the cells and activate the cognate AHL receptor (LuxR) and induce QS-regulated gene expression. (**Right**): Quorum Sensing (QS) in Gram-positive Bacteria. Gram-positive bacteria, on the other hand, use autoinducer peptides (AIPs) as signaling molecules. When they reach a certain concentration threshold, the autoinducers bind to the receptor kinase, which undergoes autophosphorylation and passes the phosphate group to the cytoplasmic response regulator. This activates the required genes in the quorum sensing regulon. The figure is partially adapted from [73].

Quorum sensing (QS) is defined as a density-dependent microbial signal system that helps bacteria perceive and respond to temporal and contiguous environments (Figure 2). Quorum sensing depends on a network of autoinducer synthases, autoinducers (AIs), partner autoinducer receptors, and downstream signal transduction components [50]. AIs are innately produced at the basal level and gradually build up as microbial growth continues, leading to a positive feedback loop [51]. With the accumulation of critical concentrations of AIs, specific receptors become activated to start a signaling cascade of coordinated induction/repression of target genes within the bacterial population. This occurs under various environmental incentives, such as morphogenesis, biofilm formation, bioluminescence, drug resistance generation, regulation of the expression of virulence factors, dormancy generation, immune escape, and others [52,53,54]. Furthermore, it is important to consider that apart from quorum sensing as the determinant of cell density, there are other environmental signals (e.g., temperature, pH, osmolarity, oxidative stress, and nutrient deprivation) that bacteria must gather information about to determine their survival strategy [55]. QS-mediated regulation of virulence determinants has been found in both Gram-negative and Gram-positive bacteria [53,56].

The discovery of the first quorum sensing system dates back to the 1970s, when *Vibrio fischeri*, a bioluminescent marine bacterium, was found to colonize, symbiotically, the light organ of the Hawaiian squid *Euprymna scolopes*, establishing a positive correlation between the bacterial population density and the expression of genes responsible for bioluminescence in the host [57,58]. For the first time in 1994, the concept of the production of signal molecules by bacteria and their subsequent release into a specific environment was proposed as quorum sensing [59]. QS plays the most important role in biofilms formed by *Salmonella* spp., *Escherichia coli*, *Campylobacter* spp., *Staphylococcus aureus*, *Listeria monocytogenes,* and *Bacillus cereus* [60]. About 80% of microbial infections have been found to be related to QS-mediated biofilm formation [61].

There is great inter-population variation in the process of sensing signals, the type of signal molecules, the receptor of signal molecules, the mechanism of signal transduction, and the ultimate phenotype [62]. The AIs produced during QS range from molecules of low molecular weight to molecules of high molecular weight, such as oligopeptides [63]. Some examples of low molecular weight molecules involved in QS are N-acylhomoserine lactone (AHL or AI-1) [64], furanosyl borate diester (AI-2) [65], 4,5-dihydroxy-2,3-pentanedione (DPD) [66], 3-hydroxypalmitic acid methyl ester (3OH-PAME) [67], *cis*-11methyl-2-dodecenoic acid (diffusible signal factor, DSF) [68], 2-isocapryloyl-3R-hydroxymethyl-*c*-butyrolactone (A-factor) [69], diketopiperazines (DKP) [70], 2-heptyl 3-hydroxy-4-quinolone [71], and 4-hydroxy 2-heptylquinoline (HHQ) [72].

## 3. Mechanism through Which Biofilms Combat Antibiotics

### 3.1. Metabolic Activity Heterogeneity and Tolerance Acquisition

The rate of metabolic activity inside a biofilm can vary significantly due to differences in the concentration of oxygen and nutrients that are available for the cells either at the surface or in the deep region of the biofilm. The bacterial subpopulations that feature the fastest growth rate are those residing on the surface of the biofilm, where oxygen availability is higher, limiting oxygen penetration to the slow- or non-growing populations occupying the inner zone of the biofilm. A study on the real-time detection of specific metabolites through fluorescent tags has revealed that the cells in the center of the biofilm are less active compared to the cells at the bulk liquid interface [74]. Another study focusing on the measurement of oxygen distribution at varying depths of biofilm using microelectrodes concluded that oxygen distribution strongly correlates with the biofilm structure and that it is depleted by as much as 30-fold in the core of the biofilm [75]. This gradient eventually leads to phenotypic and metabolic bacterial diversity, with a larger population displaying varied gene expression and phenotypes (i.e., susceptible, resistant, and tolerant cells) that co-evolve over time within the structure of the biofilm [76]. The low metabolic activity of these bacterial cells can be translated into low antibiotic target production and limited activity of such targets (i.e., enzymes involved in replication, protein formation, or peptidoglycan production). Unlike resistant cells, which, through genetic changes, develop long-term resistance to antibiotics, tolerant cells cannot grow or replicate during drug exposure but resume their growth when the antibiotic is removed. These cells are called persister cells, as they are able to outlive these unfavorable environmental conditions. This persistence is a transient phenotypic state rather than a genetic trait, as the same cells in planktonic form will become susceptible again. Persister cells are found in several human pathogens, including *Staphylococcus aureus*, *Mycobacterium tuberculosis*, *Escherichia coli*, *Salmonella enterica* subsp. *enterica* serovar Typhimurium, and *Pseudomonas aeruginosa* [77].

### 3.2. Adaptive Stress Responses

Biofilms are characterized by gradients of nutrients and oxygen that represent spatially organized stress conditions for the bacterial population, which in turn trigger adaptive responses such as the stringent response, the SOS response, and the general stress rpoS response, impairing the efficacy of antimicrobials and contributing to overall antibiotic tolerance [78]. Nutrient deficiency can also impact antimicrobial resistance as a consequence of the activation of stress responses that promote resistance by recruiting mechanisms of antioxidant activity and biofilm resistance or by modifying the cell surface to prevent the binding and entry of antimicrobials [79].

In stress conditions, when bacterial biofilms have populations with different growth rates (fast-growing, non-growing, or slow-growing cells), the availability of antibiotic targets is reduced in slow-growing cells. For example, β-lactams are effective on dividing bacterial cells only, which is evident in the large difference in minimum inhibitory concentration (MIC) and minimum biofilm inhibitory concentration (MBIC) of this antibiotic (1000 fold) in *Pseudomonas aeruginosa* [80]. Several clinically important antibiotics interfere with protein synthesis by binding at various functional centers of the ribosome, resulting in either freezing the ribosome in a particular conformation or hindering the binding of its ligands [81]. On slow-growing cells, antibiotics that bind the ribosome reversibly, such as tetracycline, have a bacteriostatic effect, while antibiotics that bind the ribosome irreversibly, like aminoglycosides, have a bactericidal effect. This selective effect of drugs on cells is due to cell growth’s dependence on ribosome abundance and protein synthesis [82]. DNA-binding quinolones are effective on non-growing cells but not as effective as on fast-growing cells [83].

The stringent response in persister cells is initiated through activation of the alarmone guanosine-5′-(tri)diphosphate-3′-diphosphate ((p)ppGpp), altering cellular physiology through transcriptional changes in populations with low metabolic activity, which consequently relocate cellular resources and ensure the survival of the bacterium [84]. Most antibiotics target active metabolic processes, and with this immediate shut-down of metabolism and growth, high levels of (p)ppGpp make bacteria insensitive to the actions of antibiotics [85]. A stringent response makes *Pseudomonas aeruginosa* biofilms tolerant to fluoroquinolones, meropenem, and gentamycin by preventing the accumulation of reactive oxidative species (ROS), which is considered a common mechanism by which antibiotics kill bacteria [86,87]. Polymyxins that target the membrane of Gram-negative bacteria are effective on non-growing populations, but metabolically active populations show adaptive resistance, impairing the penetration of the antibiotic [88]. However, the synergistic effect of combined antibiotics (tobramycin and colistin) was shown to successfully kill the metabolically active population of *Pseudomonas aeruginosa* on the surface of biofilms and the metabolically inactive population in the center of the biofilms, respectively [88,89].

Environmental stress also acts as a defining signal for recruitment of the multidrug efflux system as a stress response in many bacteria [79]. For example, the temporary activation of multidrug-resistant efflux pumps in *Pseudomonas aeruginosa* is triggered by adverse environmental conditions such as exposure to ROS (*MexXY-OprM*) [90], nitrosative stress (*MexEF-OprJ*) [91], or membrane-damaging agents (*MDAs*) [92]. An additional example is the temporary induction of β-lactamases and the activation of multidrug-resistant efflux pumps in *Pseudomonas aeruginosa* biofilms, which occurs only in the presence of β-lactam molecules. The induced β-lactamases are partially excreted by membrane vesicles into the matrix, where they inactivate β-lactam antibiotics before reaching cells [93].

The SOS response is a stress response to DNA damage that promotes tolerance to fluoroquinolones by inducing the expression of DNA repair mechanisms in *Escherichia coli* and *Pseudomonas aeruginosa* [94]. Sometimes, the common mechanism of antibiotics also triggers an SOS response in bacteria. For example, β-lactams, fluoroquinolones, or aminoglycosides all rely on the production of ROS. When ROS levels are not high enough, DNA oxidative damage (mutations) can occur, accompanied by the activation of the SOS response, repair of DNA damage, and the onset of antibiotic tolerance [95]. There is also selectivity in gene expression in planktonic and biofilm cells [96,97].

### 3.3. Antibiotic Resistance

Bacteria living in biofilms can exhibit a 10- to 1000-fold increase in antibiotic resistance as compared to similar bacteria living in planktonic states. For example, 100% of *Staphylococcus epidermidis* isolates were susceptible to vancomycin in a planktonic state, while 75% of the same bacteria were resistant when tested in a biofilm.

Many challenges are encountered by the antibiotic when it tries to penetrate the sticky, slimy membranes of the cells at the surface of biofilm. These are mostly represented by the complex biofilm architecture as well as the extracellular proteins and eDNA, which prevent the antibiotic from reaching its target. Meanwhile, the antibiotic may be deactivated even before it reaches its target. At the level of the microenvironment, metabolic byproducts, waste, and nutrients have started to accumulate, and oxygen supply from the surface may be greatly reduced, creating an anaerobic environment. The combination of all these factors can affect antibiotics in different ways, depending on their chemical structure and mechanism. For example, low oxygen levels reduce the bactericidal effects of tobramycin and ciprofloxacin, while pH changes can negatively influence aminoglycoside action. In such a situation, the resistant cells deep inside the biofilm enter the dormant state (persister cells), in which cell division is avoided. This preservation mechanism protects bacteria from the action of antibiotics, which usually require cells to be actively dividing. Interestingly, this dormancy is not permanent and reverses back to normal once cells are released from the biofilm [49].

### 3.4. Horizontal Gene Transfer (HGT)

Inside biofilms, horizontal gene transfer (HGT) is one way of driving the spread of antibiotic resistance genes (ARGs) due to the restricted motility of cells embedded in a matrix and eDNA as a means of intercellular contact. Moreover, as compared to natural transformation (transfer of chromosomal DNA and non-conjugative plasmids) and bacteriophage infection (transfer of bacteriophage genomic DNA), conjugation (transfer of conjugative plasmids and of integrative and conjugative elements (ICEs)) is the most common HGT mechanism in biofilms [98]. Due to their spatial organization, only those cellular subpopulations that reside in the core of biofilm can undergo HGT. However, biofilm growth promotes the persistence of plasmids carrying resistance genes. For example, in *Staphylococcus aureus* biofilms, conjugative plasmids were found to be 1600 times higher than in planktonic cultures. Another way of achieving HGT in Gram-negative bacterial biofilms is through integron-mediated acquisition/exchange of antibiotic resistance determinants by specific regulation of class 1 integron integrase [99,100].

### 3.5. Efflux Pumps in Biofilm Resistance

Efflux pumps are transport proteins involved in the removal of different metabolites, including antibiotics and secondary metabolites, to avoid toxic accumulation. They are thus implicated in antibiotic resistance and may promote biofilm antimicrobial resistance in several bacterial species [101], including *Burkholderia cenocepacia* [102], *Escherichia coli* [103], and *Pseudomonas aeruginosa* [104]. About twelve resistance-nodulation-division (RND) families of efflux pumps have been identified in *Pseudomonas aeruginosa*, four of which mediate antibiotic resistance [105]. *MexAB-OprM*, one of many efflux systems in bacteria, is most closely related to carbapenem resistance in *Pseudomonas aeruginosa* [106]. The regulatory genes *mexR*, *nalD,* and *nalC14* negatively regulate the expression of *MexAB-OprM*, and any type of mutation in them may lead to the upregulation of *MexAB-OprM*, resulting in increased drug resistance in *Pseudomonas aeruginosa* [107].

## 4. Biofilm Disruption Strategies

Since biofilm formation contributes to bacterial pathogenicity and antibiotic resistance, various strategies have been employed to deal with this problem encountered in biofilm-infected tissues, tissue implants, and medical devices (Table 1).

### 4.1. Anti-Adhesion Strategies

Using anti-adhesion strategies, the exterior surface of the implanted medical device or biomaterial is altered, either directly or with the aid of a coating, to create a barrier that is not supportive of bacterial adhesion. Bacteria use cell appendages like pili or flagella along with physical factors like van der Waal’s forces, Brownian motion, or electrostatic interactions to adhere to biotic and abiotic surfaces. To colonize a host tissue surface, bacteria can adhere to host-produced components like fibrinogen, fibronectin, collagen, fibronectin-binding proteins (FnBPs), and fibrinogen-binding clumping factors (Clfs) [152].

Likewise, hydrophobic and non-polar surfaces also facilitate microbial binding. Coating the surfaces with antimicrobial agents such as metal-based nanoparticles, grafting the surface with cationic polymers, fabricating bionic antibacterial surfaces with a nano-scale structure, and using surfactants are good strategies to prevent bacterial adhesion on various surfaces. The strategy of inhibiting bacterial adhesion has been helpful in preventing biofilm-related infections on orthopedic implants [153]. Another study used glass, stainless steel, and silicon surfaces pretreated with dicephalic quaternary ammonium salts (QAS) to limit the adhesion of *Staphylococcus epidermidis* and *Candida albicans* cells [154]. Despite remarkable developments, anti-adhesion surface approaches featuring long-term stability need further investigation [153].

### 4.2. Quorum Quenching or Quorum Sensing Inhibition

QS regulates the expression of bacterial virulence factors, and therefore blocking QS can curb the virulence of bacteria. Novel therapeutic approaches interfering with QS, termed quorum sensing inhibition (QSI) or quorum quenching (QQ), have been introduced [155]. QS inhibitors (QSIs) interact with QS signaling systems in several ways, including (i) inhibition of synthesis; (ii) degradation; (iii) competition for receptor sites; (iv) inhibition of gene expression; and (v) removal of AIs. Since QSIs do not work through bactericidal or bacteristatic mechanisms to reduce bacterial virulence and biofilm formation, they pose less pressure for resistance selection in bacteria [156]. QSIs and quorum quenching (QQ) enzymes are the main QS inhibitors, and their functional targets include QS signaling molecules, receptors, and downstream signaling cascade components [157].

#### 4.2.1. Targeting QS Signaling Molecules

Blocking the synthesis of QS molecules (AHLs and AIPs) in Gram-positive and Gram-negative bacteria or degrading them results in QS inhibition. AHL-lactonases, oxidoreductases, and antibodies are the major QS inhibitors. They target AI signaling molecules by disabling the enzymes that are responsible for their synthesis.

AHL-lactonase and AHL-acylase hydrolyze the lactone ring of AHL or cleave the acyl side chain of AHL in Gram-negative bacteria, which reduces AHL-LuxR binding and thus curbs QS signaling [158]. In Gram-negative bacteria, N-acyl homoserine lactone oxidoreductase, another QQ enzyme, modifies AIs and hinders their specific binding to receptors, resulting in reduced biofilm formation [138].

It has also been reported that antibodies play a role in the inhibition of QS signaling molecules [138]. In this regard, AIPs, which are produced by Gram-positive bacteria, are susceptible to antibody neutralization, as AHL bacterial molecules act as small-molecule toxins in mammalian cells, resulting in apoptosis and modulating NF_-_κB activity. For example, the XYD-11G2 antibody prevented the production of pyocyanin by *Pseudomonas aeruginosa* and neutralized the 3-oxo-C12-HSL signal [159]. A study on AIP-4 produced by *Staphylococcus aureus* observed its effective blocking by an anti-AI monoclonal antibody (AP4-24 H11) [160]. In addition, several naturally occurring brominated furanones have the ability to inhibit the LuxS enzyme in a concentration-dependent manner [161]. There are many reviews on QS molecules as targets for biofilm disruption [162,163].

#### 4.2.2. Targeting Signaling Molecule Receptors

Inhibiting or competing for QS receptors is another strategy used by QS inhibitors. LuxR-AHL is a significant AI receptor protein found in Gram-negative bacteria. Therefore, disrupting the bond between signal receptors and AHLs with AHL analogs, structurally independent AHLs, and naturally occurring QS inhibitors is an effective alternative strategy for controlling QS. Several bulky groups have been added to the acyl side chain to create AHL analogs in *Pseudomonas aeruginosa*, *Agrobacterium tumefaciens,* and *Vibrio fischeri*, respectively, which have demonstrated the inhibition of LasR, TraR, and LuxR receptors [164].

Two types of naturally effective QS inhibitors are furanones and flavonoids, which can bind to the receptors of many pathogenic bacteria. Furanones are produced by the red alga *Delisea pulchra* and are known to regulate bacterial colonization and biofilm development through interference with the acylated homoserine lactone regulatory system in Gram-negative bacteria and the alternative AI-2 signaling system in Gram-negative and Gram-positive bacteria. Many furanones are now known as competitive inhibitors of LuxR-type receptors in Gram-negative bacteria by competing with AHL for binding to reduce QS signaling. Natural furanone ascorbic acid (vitamin C) is known to be a potent inhibitor of QS in *Pseudomonas aeruginosa* [165]. It has been shown to inhibit pyocyanin production, which supports cellular respiration and energy generation in oxygen-deficient conditions in *Pseudomonas aeruginosa* biofilms, thus affecting biofilm formation [166]. Libraries of synthetic furanones have also been developed, as they are potent anti-infectives and inhibit pathogenic phenotypes in Gram-negative and Gram-positive bacteria [167]. Flavonoids (e.g.,quercetins) are other natural QS inhibtors that are found in various plant parts (flowers, leaves, seeds) [168]. Quercetins are effective QS inhibitors in *Pseudomonas aeruginosa*, as they can inhibit biofilm formation and initial bacterial adherence and reduce virulence factor expression by competing with AHL for binding to the LasR receptor [169].

Another target for receptor binding competition is the competence stimulating peptide (CSP)-mediated QS system in *Streptococcus pneumoniae*, which uses two main CSP variants: CSP1 and CSP2, which bind to their corresponding histidine kinase receptors, ComD1 and ComD2, resulting in virulence and biofilm formation. Synthetic peptides like dominant-negative competence-stimulating peptides (dnCSPs) that compete with CSP for ComD binding have been used to reduce virulence factor expression in vitro and attenuate pneumococcus infections in mice [170,171]. To target the AI-2 QS system in Gram-positive bacteria, sulphone is among the many compounds that have shown an antagonistic effect on LuxP receptors in *Vibrio harveyi* [172]. There is detailed discussion of inhibitors targeting QS signal molecule receptors in several reviews [73,173,174].

#### 4.2.3. Blocking the Signaling Cascade

Blocking the signaling cascade by deactivating the downstream response regulators or other regulatory factors is another strategy for QS inhibition. For example, triggered by upstream signaling, the downstream response regulator *AgrA* of *Staphylococcus aureus* is activated to induce the expression of QS-related genes. In this regard, Savarin, a known *Staphylococcus aureus* virulence inhibitor, can specifically target *AgrA* to stop the signaling cascade [73].

Virstatin, a small molecule, represses the expression of *AnoR*, which positively regulates LuxI-like synthase AnoI in *Acinetobacter nosocomialis.* This results in a reduced production of N-(3-hydroxy-dodecanoyl)-L-homoserine lactone (OH-dDHL), thus affecting the signaling cascade and reducing biofilm formation and motility [175]. There are several reviews on QS interfering mechanisms and their implications for bacterial pathogenecity [157,173,176].

#### 4.2.4. Targeting the EPS Chemical Composition and Structure

There are several ways to target EPS matrix formation, mostly related to the inhibition of EPS production via the prevention of adhesin-mediated bacterial attachment to surfaces or to the degradation of EPS matrix in mature biofilms using mutants of enzymes that are produced by the bacteria themselves [177]. Despite being considered biofilm virulence factors, these enzymes can be engineered to initiate biofilm disassembly. Enzymes like glucano-hydrolases and glycoside hydrolases disrupt the viscosity and elasticity of the biofilms, which weakens biofilm cohesiveness and increases antibiotic penetration [178].

eDNA was found to play a vital role in the composition of bacterial biofilms in the context of HGT, and this paved the way to targeting eDNA with DNases. Dnase-I destroys eDNA through the hydrolysis of its phospholipid ester bonds [179]. Exogenous DNase I has shown inhibition of biofilms of many Gram-negative and Gram-positive bacteria, but it is more effective on young biofilms [179,180].

Bacteriophages have the ability to penetrate the tridimensional architecture of biofilms and eradicate bacterial biofilm. The novel phage F15 produces a polysaccharide depolymerase that hydrolyzes the EPS of *Pseudomonas putida* and inhibits biofilm formation [181]. To maintain biofilm adhesion properties and stability, extracellular proteins like DNA-binding proteins (DNABPs), functional amyloids/amyloid-like proteins (FA/ALPs), and other biofilm-associated proteins (Baps) are crucial [182]. Hence, proteases (e.g., Purified Esp [183], proteinase K [184], and cysteine proteases [185]) that can degrade EPS extracellular proteins have the potential to disperse a massive biofilm [177]. Recent research has proven curcumin, a distinctive yellow pigment and a major constituent of turmeric derived from the Curcuma longa plant, to be a potent anti-QS agent in many pathogens as it inhibits the production of QS-dependent factors such as exopolysaccharide and alginate [186]. In general, the combination of EPS synthesis inhibitors or EPS-degrading enzymes, which lack intrinsic antibacterial activity, with antimicrobial agents could be a good option for biofilm removal [187]. Many reviews on EPS degradation and synthesis inhibition have discussed this strategy in detail [188,189].

### 4.3. Targeting Persister Cells

There are several strategies to kill persister cells in biofilms: (1) the direct killing of metabolically dormant persister cells; (2) awakening the persister cells from metabolically inactive form to antibiotic-susceptible active form; (3) combining anti-persister drugs with conventional antibiotics; and (4) other indirect approaches such as interfering with the QS signaling circuit and genetic engineering of the metabolic pathways of persister cells [190]. Anti-persister agents, such as cationic antimicrobial peptides (AMPs), make pores on respiring cells as well as persister and dormant populations residing in the center of biofilms. They change the membrane potential through electrostatic interactions with oppositely charged cell membrane/wall components. A broad-spectrum antimicrobial peptide, TM5, can reduce planktonic and persister cells in biofilms formed by both Gram-positive and Gram-negative bacteria, but its in vivo clinical potency needs validation [191]. Arginine and tryptophan-containing cationic membrane-penetrating peptides have been shown to destroy the negatively charged lipopolysaccharide of the persistent *Escherichia coli* cell wall, resulting in membrane disruption and cell death [192]. Since AMPs have shown more biofilm inhibition than eradication potency, they have now been used in combination with antibiotics.

### 4.4. Targeting Efflux Pumps

Besides being a major factor in the development of antibiotic resistance, efflux pumps can influence the functions of biofilms directly or indirectly [193]. Upon exposure to tigecycline, *Acinetobactor baumannii* was shown to feature an attenuated tendency to form biofilm due to the downregulation of the *adeG* gene encoding for efflux pumps [194]. Many studies suggest that the bacterial QS mechanism is negatively affected if inhibitors hinder the efflux pump activity [195]. Another study on *Acinetobactor baumannii* demonstrated that with inhibition of the *adeAB* gene (for the MDR efflux pump belonging to the RND family) expression or deletion, hindrance of biofilm and QS systems occurs [196]. Chetri et al. [197] have thoroughly discussed the urgent need for improved efflux pump inhibitors.

## 5. Light-Based Antibiofilm Strategies

Owing to the high spatio-temporal control with which a light stimulus can be delivered, light-based strategies against bacterial proliferation and biofilm formation represent a rapidly expanding field of science. Compared to traditional pharmacotherapy, this distinct advantage makes light the perfect stimulus to confine the antibacterial action only when and where it is needed. Interestingly, light-triggered approaches against bacterial proliferation have been reported in the context of all developmental stages, starting from adhesion to biofilm formation, initiation, and maturation.

### 5.1. Bacterial Adhesion: Light-Triggered Control of Bacterial Adhesion

Smart antibacterial surfaces have been investigated for decades due to their potential to control bacterial attachment, biofilm formation, and dissolution. In 2018, Li and co-authors published a comprehensive review focusing on the design of smart antibacterial surfaces with antibiofouling and antimicrobial properties [28]. The idea behind the design of a smart surface with antimicrobial properties is to reduce the development of drug-resistant bacteria by means of a local and on-demand administration of the drug or to avoid bacterial adhesion and therefore biofilm formation. Different stimuli have been used to achieve controlled antimicrobial drug release, including pH, temperature, release of chemicals, use of charged polymers, and nano- and microstructured surfaces with biomimetic properties [198,199,200,201,202].

**Figure 3:**
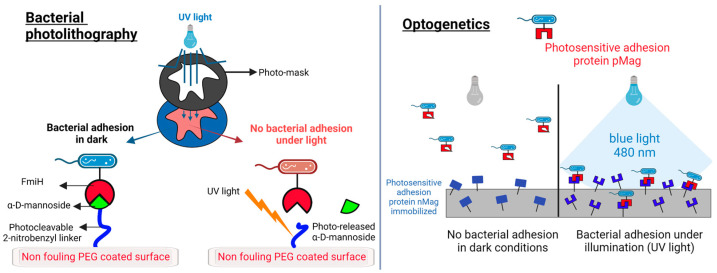
Light-triggered strategies against bacterial adhesion. (**Left**): the photolithography approach to controlling bacteria adhesion [203]. The α-D-mannoside is immobilized on a PEG surface by means of a photocleavable 2-nitrobenzyl linker. The adhesive protein FimH in *Escherichia coli* binds to the sugar in dark conditions. Under UV light, the linker is cleaved, the sugar is released, and the surface becomes antiadhesive for the bacteria. The patterning of illuminated and non-illuminated regions can be as small as 10 µm. (**Right**): the optogenetic approach to controlling bacterial adhesion. *Escherichia coli* bacteria express on their surface a photoswitchable protein (pMag) that can heterodimerize with the nMag protein immobilized on the surface under blue light (480 nm). In dark conditions, the binding is impeded, and no bacterial adhesion can be obtained. Binding is reversible and can be repeated by illumination and dark cycles. The scheme is adapted from Chen et al. [208].

In this review, we make reference to those applications in which the antibacterial action is triggered by light. In this regard, the light stimulus can be addressed to control surface functionality, such as bacterial photolithography, or to control the bacteria’s adhesion properties, such as in optogenetic approaches (Figure 3).

Bacterial photolithography allows the control of biofilm patterning at a distance as small as 10 µm [203]. For biotechnological applications or to study complex bacterial communication circuits such as those involved in quorum sensing, the goal is the controlled patterning of the biofilm rather than its total eradication. Indeed, obtaining patterned biofilms on the microscale can allow the investigation of bacterial communication and biofilm formation [204], the exploration of the use of biofilms as living biomaterials [205], and the generation of more reliable results of drug tests due to the more controlled biofilm geometries [206]. A bottom-up approach to control bacteria colonies has been proposed. It involves a genetically-encoded biofilm patterning tool named “Biofilm Lithography”, which is able to control the expression of membrane adhesion proteins that are responsible for surface attachment [204]. This allowed the patterning of *Escherichia coli* biofilms with a 25-micrometer spatial resolution.

With the aim of patterning biofilm, Chen et al. [203] used a photolithography approach with *Escherichia coli* bacteria. The surface was functionalized with a mannoside group on a nonadhesive polyethylene glycol (PEG) coating (Figure 3, left-hand side). The α-D-mannoside group is recognized by the FimH bacteria receptor, and bacteria can adhere to this functionalized surface. The mannoside group is connected to the PEG coating by means of a photocleavable 2-nitrobenzyl linker. Upon exposure to UV light, the linker is cleaved, and the nonadhesive PEG becomes exposed. Photopatterning allows bacteria to adhere in non-illuminated regions and prevents them from adhering in illuminated regions and on bare PEG surfaces.

Sugar binding strategies were also used by Ma et al. [207], who developed a spiropyran- and galactose-decorated nanoplatform. These interactions were developed to image bacterial adhesion and eradicate the *Pseudomonas aeruginosa* biofilm from the surface.

Bacterial binding properties can be controlled with an optogenetic approach. This technology is well established in neuroscience and provides interesting opportunities to control bacterial behavior at the cost of introducing genetic modifications in the pathogen [204,208,209,210,211].

Regarding the possibility of controlling bacterial adhesion and biofilm organization, Chen et al. [208] proposed the use of photoresponsive proteins (nMag and pMag), which heterodimerize under blue light (480 nm) and dissociate from each other in the dark. The author expressed pMag on the surface of *Escherichia coli.* This protein can interact under blue light with nMag immobilized on a glass substrate coated with PEG. In the dark, the adhesion is reversible. This allowed for blue-light switchable bacterial adhesion with high spatial and temporal resolution (Figure 3, right-hand side).

This optogenetic control has been recently scaled up from the surface of the material to the dynamically controlled bacteria-bacteria adhesion [212]. The ability to control photoswitchable adhesion between bacteria is important to regulate multicellular and associated bacterial behaviors such as aggregation, quorum sensing, biofilm formation, and metabolic processes. Chen et al. obtained this result by expressing pMag and nMag proteins on the surface of *Escherichia coli* to obtain bacteria that cluster when illuminated with blue light and disassemble in the dark.

Notably, in contrast to the photolithographic approach, which is not reversible as it involves the photocleavage of the linker on the surface, the optogenetic approach allows a reversible control on the bacterial adhesion since the adhesion can be repeatedly turned on and off.

### 5.2. Bacterial Communication: Photoswitchable Modulators of Quorum Sensing

As detailed in Section 2, quorum sensing is the communication system that bacteria use to organize into communities and develop biofilms. QS has been considered a target for synthetic biology strategies to control biofilm development [213]. In this regard, the possibility of using light to control and interfere with this communication system is extremely appealing. A tool that is able to control QS offers the possibility to control bacteria group biology, study QS circuits, inhibit biofilm formation, or promote biofilm dispersal on command, if needed [214]. The Feringa group investigated the possibility of using photoswitchable compounds to interfere with QS. Their molecular design relied on the modification of autoinducer molecules via a light-sensitive moiety. The introduction of an azobenzene unit in the N-Acyl homoserine lactones (AHLs) led to a small library of photoswitchable autoinducers for Gram-negative bacteria [215]. Their design, which was supported by previous SAR data and computational pharmacophore models of the AHL [164], led to the replacement of the alkyl chain of the lead compound with the azobenzene moiety. By means of bioluminescence assays measuring the LasQS-controlled bioluminescence in *Escherichia coli*, they obtained the opposite behavior for two of the tested molecules. Indeed, one of the molecules acquired QS-inducing activity while the other lost its activity upon *trans-cis* isomerization, proving that the photocontrol of the QS mechanism in bacteria is possible and that the contradictory behavior under light illumination can be ascribed to the geometry of the molecule. The active molecules have a more linear shape and therefore a better interaction with the receptor binding pocket than the less active conformations, which are more bent. Finally, by acting on the LasQS system, the authors were also able to photocontrol the production of virulence genes in *Pseudomonas aeruginosa* [215]. The gene expression of *lasA* was different for the irradiated and non-irradiated compounds, proving that it was possible to control the expression of virulence genes with light.

**Figure 4:**
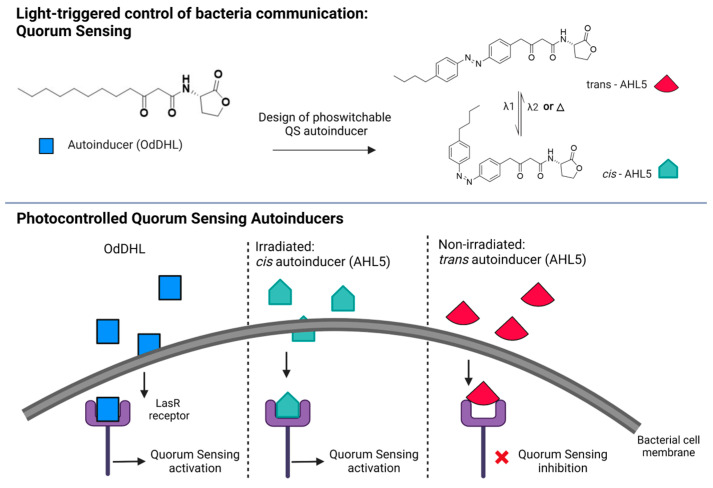
Photoswitchable modulators of bacterial communication. Top panel: native QS autoinducer (OdDHL) and photoswitchable analogue design (AHL5) in trans and cis conformation. *Trans*-AHL5 is the thermodynamically stable form of the molecule, which can be switched to *cis*-AHL5 by UV light illumination (λ_1_ = 365 nm). A backward reaction from *cis* to *trans* can be obtained by exposure to visible light (λ_2_) or heat (Δ). Bottom panel: scheme of action of the autoinducer binding and activating the LasR receptor that triggers QS (native OdDHL and *cis*-AHL5) or acting as an antagonist (*trans*-AHL5) and inhibiting the receptor [216].

A recent follow-up study from this group [216] led to the development of photoswitchable autoinducers in *Pseudomonas aeruginosa* based on N-3-(oxo-dodecanoyl)-L-homoserine lactone (OdDHL) (Figure 4 on the top). In a bioluminescence assay in a QS reporter *Escherichia coli* strain, the authors evaluated the agonist or antagonist character of a library of photoswitchable compounds. One of the best-working compounds (AHL5) (Figure 4, bottom) showed a remarkable 700-fold difference in activity between the two forms, with a switching behavior between antagonist (non-irradiated) and agonist (irradiated with 365 nm) at 60 µM. This makes these compounds good candidates for QS studies.

### 5.3. Biofilm Maturation and Planktonic Phase: Photocleavable and Photoswitchable Antibiotics

**Figure 5:**
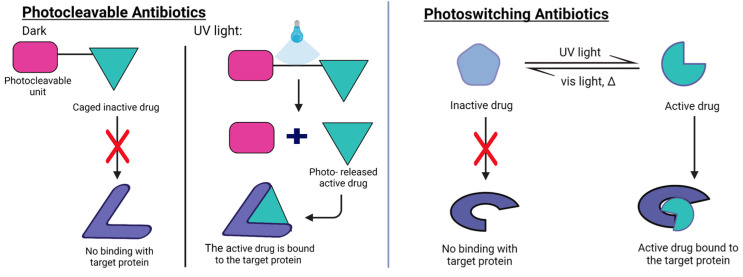
Mechanisms of action of photocleavable antibiotics (**Right**) and photoswitchable antibiotics (**Left**). Photocleavable antibiotics consist of a drug molecule that is inhibited by the presence of a photocleavable moiety. Upon light-triggered cleavage of the impeding unit, the free antibiotic can interact with the target protein. Photoswitchable antibiotics feature a bistable form where only one of the conformations can interact with the binding protein and exert the pharmacological action. This strategy gives reversible control over the pharmacological action of the antibiotic, which can also be deactivated by switching back the molecule to its inactive form, either spontaneously with time or by using an appropriate wavelength of light.

Two different strategies have been implemented to develop light-triggered antibiotics: (i) photocaged compounds and (ii) photoswitchable compounds (Figure 5). The molecular design is intrinsically different and has been recently reviewed [202]. In photocaged compounds, a photolabile moiety is attached to the drug scaffold, hindering its interaction with the biological target. This photolabile moiety is then removed by illumination, thus letting the drug interact with the biological target.

The design of photoswitchable antibiotics involves the inclusion of a photoswitchable unit permanently attached to the antimicrobial drug. While, under dark conditions, the interaction with the biological target is hindered, the photoswitching of the light-sensitive moiety produces a conformational change that favors the interaction with the biological target. For photoswitchable antibiotics, the presence of a photoswitching unit can cause a reduced interaction with the biological target even in the case of the best performing isomer, thus decreasing drug potency. On the contrary, the presence of a photolabile element in caged antibiotics allows the release of the intact drug. However, the irreversibility of the photochemical reaction makes it impossible to deactivate the action of those drugs once the caged unit has been released. Photoswitchable antibiotics have, instead, the advantage that if the metastable form (i.e., the *cis* form) is the pharmacologically active conformation of the compound, it will deactivate spontaneously after the compound converts back to the thermally stable form (i.e., the *trans* form). This confers on the photoswitchable antibiotic a lower impact on the environment and a lower risk of triggering antibiotic resistance in bacteria.

The possible mechanisms of AMR development from photoswitchable drugs are also being studied. A 2021 study [217] analyzed the development of resistance in the *Escherichia coli* mutant strain CS1562 by the effect of *trans*/*cis*-tetra-ortho-chloroazobenzene-trimethoprim (TCAT) compounds. Both irradiated TCAT and thermally adapted TCAT were analyzed and compared to the reference analog trimethoprim (TMP). Interestingly, the photoswitchable compound had a different response to acquired resistance. The resistance mechanism to the photoactive compound appears to hinder the entry of the molecule into the cell, whereas the resistance to TMP involves changes in cell metabolism and alterations in the expression levels of enzymes associated with the biosynthesis of folate.

Hou et al. [218] developed CONBE, a photolabile ciprofloxacin compound. Their design strategy involved the conjugation of a photocleavable ortho-nitrobenzene to the 3-carbonyl of ciprofloxacin. After illumination with UV light (365 nm, 10 mW/cm^2^), leading to the photochemical cleavage of the cage element, the conversion of CONBE to ciprofloxacin was 91% efficient. The bactericidal activity obtained with this strategy was as potent as that of the parent drug. A light-activatable caged antibiotic based on vancomycin and cephalosporin was proposed by the Gademann group [219]. The photoreleased vancomycin was active against Gram-positive strains, and the uncaged cephalosporin was active against both Gram-positive and Gram-negative strains. To overcome the problem of using phototoxic UV light, vis-NIR activatable cleavage groups can be used. Contrera-Garcia et al. [220] proposed the design of a BODIPY photocage to protect quinolone-based antimicrobial compounds. This design strategy allowed the authors to use green (λ_max_ = 520 nm), red (λ_max_ = 635 nm), or far red (λ_max_ = 730 nm) light for the photorelease.

Regarding photoswitchable antibiotics, their molecular design has been extensively reviewed in recent times [202]. Light-sensitive analogues of natural compounds, cystobactamids, have been proposed. Cystobactamids are active against a broad range of Gram-negative and Gram-positive pathogens by targeting bacterial gyrase [221]. Due to poor light penetration into biofilm colonies, the use of these compounds against the biofilm state is still difficult, and most of the studies focus on planktonic life styles. Researchers are exploring different chemical designs to gift azobenzenes with red and infrared absorption to allow antibiotic activity with deeper penetration [222].

Photosensitive surfactants based on photoswitchable compounds such as AzoTAB have been studied for their ability to perturb a lipid membrane and cause the disruption of vesicles [223]. This strategy can provide engineered compounds to target the bacteria’s membrane. This would produce photoswitchable antibiotic agents that reside in the membrane and, when activated by light, can cause its disruption, thus leading to the death of the bacteria [224]. An elegant evolution of this concept, albeit on cancer cells, has been proposed by Mutter et al. [225]. Fragaceatoxin (FraC) is a toxin able to form nanopores on the surface of sphingomyelin-rich cells, causing cell death. FraC has been decorated with azobenzene molecules with the aim of controlling the nanopore assembly with light. The authors obtained a system that is inactive in the dark but active upon illumination, causing cell lysis.

Finally, new targets for photoswitchable compounds have been explored, such as bacterial motility and bacterial membrane potential. Duchesne et al. [226] applied photoswitchable compounds to control the speed of *Escherichia coli* with light. They obtained different net effects (increase or decrease of speed) depending on the compound tested, proving that the design of such optical tools is very promising. De Souza-Guerriero et al. [227] explored the possibility of using a membrane-targeted azobenzene to photo-modulate the membrane potential in Gram-positive cells (*Bacillus subtilis*). These studies open the way to the use of optical tools to study bacteria spreading and biofilm electric signaling and develop new light-triggered strategies for bacterial infections and antimicrobial resistance.

## 6. Light-Based Materials Strategies to Tackle Bacterial Infections

A promising approach to counteracting bacterial adhesion and growth relies on the design of advanced functional materials, which may not only overcome some of the limitations encountered with photo-pharmacology strategies but also allow for a therapeutic effect without further reinforcing AMR mechanisms. The so-called “nanobiotics” harness the unique potential of their nanometric structure, shape, and tailorable surface chemistry to actively hinder bacterial processes and interfere with biofilm formation and adhesion [27,228]. Moreover, if loaded with bioactive compounds, such as bactericidal drugs (i.e., antibiotics and antimicrobial peptides) or biofilm dispersants (i.e., EPS enzymes and nitric oxide compound generators), the nanometric formulations grant a controlled and localized delivery of the cargo molecules, thus reducing the toll of potentially negative systemic side-effects [229].

Antibacterial materials are typically classified according to the stimulus that triggers their response, which can be either endogenous or exogenous. Endogenous stimuli include the acidic pH of the bacterial microenvironment and the secretion of bacteria metabolites and enzymes (such as lipases, proteases, and matrix metalloproteinases), while exogenous starters are generally physical-based, such as light, temperature, electricity, magnetic field, ion concentrations, and ultrasound [26,228]. Using light as a source of activation appears to be one of the most promising strategies, as several classes of organic and inorganic materials can respond to a broad spectrum of wavelengths. Moreover, light is not harmful to both humans and the environment; it allows a non-invasive, on/off regulation of the material’s properties and activity, and it can easily permit the control of the irradiation site and dosage [26].

**Figure 6:**
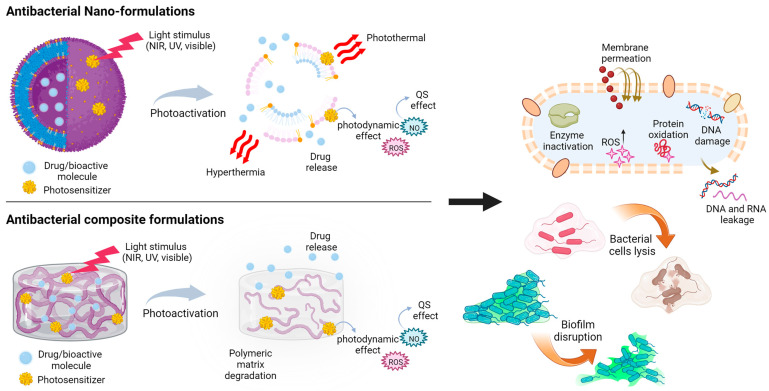
Light-triggered formulations and their antibacterial effects. The photoactivation of antibacterial functional materials can spark a series of mechanisms, such as localized and controlled drug release, photodynamic effect, and photothermal effect, which can either act on each bacterial cell individually (via DNA damage, enzyme inactivation, protein oxidation, and cell lysis) or on the bacterial biofilm. Upon light stimulation, hybrid organic/inorganic composite formulations (here depicted as composite hydrogel as an example) may degrade or modify their matrix properties and release antibiotic cargos or photodynamic nanoparticles.

Various light-activated antibacterial mechanisms have been proposed in the literature, encompassing permeation of the bacterial membrane, inhibition of enzyme activity, as well as the generation of reactive oxygen species (ROS), which in turn are responsible for toxic intracellular oxidation cascades, ultimately leading to DNA leakage, microorganism cell lysis, and biofilm disruption (Figure 6) [27,228]. Overall, the antibacterial strengths of those advanced, light-triggered material formulations reside in their ability to (i) respond on demand upon a specific luminous trigger; (ii) potentially leverage on multiple stimuli and activate distinct bacteriotoxic modus operandi; and (iii) physically breach crucial biological processes. On the other hand, conventional drugs are designed to interfere at a molecular scale but in a rather uncontrolled, systemic way, which, as a detrimental consequence, may favor the establishment of AMR.

Depending on the targeted microorganisms or biomedical applications, a plethora of antibacterial light-triggered functional materials have been developed [230], either in the form of nanoformulations [26,229,231], functionalized surfaces [28,232], or hydrogel or polymer-based formulations [25,29].

### 6.1. Light-Triggered Nano-Formulations

Depending on the intended microorganisms or biomedical applications, a plethora of antibacterial light-triggered functional nanoformulations have been developed, such as micelles [233], liposomes [234], carbon quantum dots [235,236], silver- and gold-based nanoparticles [237], mesoporous silica particles [238,239], metallo-organic frameworks (MOFs), metal oxide-based nanostructures [240], up-conversion nanoparticles [241,242], and polymeric nanoparticles [243], either loaded with antimicrobial drugs or presenting at their surfaces an optically active compound. Table 2 reports an overview of some of the most recent studies, showing the material constituents, the light stimuli that trigger their mechanisms of action, as well as the targeted microorganisms and applications.

What makes nano-formulations more versatile compared to traditional drugs or novel photo-pharmaceuticals is the different types of light sources that can be employed to prompt a specific antimicrobial mechanism, which span from near-infrared (NIR, 750–950 nm) to UV light (290–400 nm), passing through visible light (400–750 nm), and LED or laser sources. The light stimulus may either activate a nanoparticle photoactive core or trigger the plasmonic photothermal effect, which eventually leads to the generation of heat, a temperature increase at the nano-formulation surface and within its surroundings, and a necrotic cascade in the adjacent bacterial cells.

The plenitude of light-based stimuli may favor applicability in different settings, from the clinical/surgical environment to the point-of-care, personalized system. The presence of a high superficial area allows surface functionalization with photosensitizers able to enhance light absorption as well as chemical modification with targeting moieties useful for enzyme recognition or bacterial membrane/biofilm penetration. Moreover, the assorted shapes and sizes that can be easily obtained with the nanostructures can be leveraged to further widen the field of applications or the fabrication of multiple-responsive systems [256].

**Figure 7:**
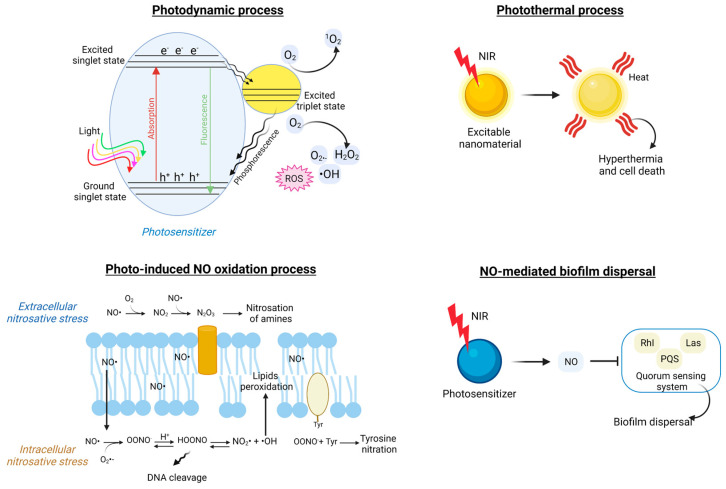
Principal mechanisms involved in light-triggered formulations antibacterial action. The photodynamic process relies on the use of photosensitizers to produce cytotoxic ROS under visible, UV, or NIR light sources and in the presence of O_2_ (schematic liberally inspired from Wang et al. [231]. Upon light irradiation, electrons are promoted from the valence band to the conductive band, leaving positive holes behind. Both charge carriers can undergo energy transfer reactions with either water molecules or molecular O_2_ in their surroundings. The photothermal effect occurs as a result of NIR light absorption by atoms at the surface of conductive nanostructures, which convert the energy into heat, ultimately causing bacterial cell death. Photo-induced NO oxidation antibacterial mechanisms include extracellular and intracellular nitrosative stresses as a consequence of the production of HOONO, N_2_O_3_, and NO_2_^●^, which eventually lead to DNA cleavage, membrane lipid peroxidation, and membrane protein nitration (schematic adapted from Carpenter and Schoenfisch [288]). When released upon NIR stimulation from a photosensitizer precursor, nitric oxide has been shown to interfere with the quorum sensing system, promoting biofilm dispersal (schematic adapted from Zhao et al. [289]).

For example, Zhao and colleagues [233] developed NIR-activated liposomes composed of a thermosensitive phospholipid, distearoyl phosphatidylcholine (DSPC), and a quaternized cholesterol molecule, which were loaded with Tobramycin and functionalized with a cyanine dye (Cypate). Upon irradiation with NIR, the susceptible dye generated a temperature increase; when reaching 45 °C, the structure of the micelle was partially disrupted, and a localized antibiotic release of up to 80% could be obtained. Moreover, the heat generation mediated by the Cypate molecule induced enzyme denaturation, leading to cell death. The synergistic action between the photothermal therapy and the on-demand drug release caused a 7- to 8-fold increment in biofilm dispersion rate relative to the conventional, free antibiotic therapy. The photothermal effect (Figure 7), which is based on the absorption of light by electrons at the surface of conductive or thermo-responsive materials and on the consequent energy dissipation in the surroundings as heat, is widely used to liberate bioactive molecules from their vehicles or to cause permanent DNA damage via hyperthermia and bacterial cell lysis [228,241].

Several antibacterial nanostrategies are based on the use of metal oxide nanoparticles as photocatalytic agents to trigger extracellular and intracellular oxidation reactions. Bagchi and co-workers [240] presented a hybrid nanosystem composed of ZnO nanoparticles decorated with a photosensitive dye (squaraine, SQ) as an antibacterial coating for artificial implants. The well-known antimicrobial activity of ZnO nanostructures resides in the light-mediated electron transfer from the valence band to the conduction band of the material and the consequent generation of electron-hole pairs. These react with oxygen molecules in the surroundings and generate reactive oxygen species (ROS) such as hydroxyl radicals (●OH), superoxide anions (O_2_^−^), and singlet oxygen (^1^O_2_), which spark oxidation cascades, intracellular protein oxidation, bacterial membrane permeation, and genetic material leakage (Figure 7) [236,257]. The use of SQ in the hybrid construct is responsible for the interfacial electron transfer and more intense ROS generation (with treatment for 3 h at a NP concentration of 140 nM). Thanks to their nanometric size (~24 nm), SQ-ZnO NPs can be internalized in *Staphylococcus aureus* cells, disrupting the bacterial membrane and reducing biofilm adhesion. Nano-formulations that present dual photodynamic and photothermal effects to enhance their antimicrobial action have been extensively proposed in the literature (Table 3) [236,242,249,250].

**Table 3:** Light-triggered anti-bacterial polymer-based or composite formulations.

Materials	Formulation	Light	Mechanism	Target Microorganism	Application
PVA-Prussian blue nanoparticle hydrogel films	Nanoparticles in hydrogels	NIR	Localized photothermal therapy	*Pseudomonas aeruginosa* [258]	
Sodium alginate hydrogel loaded with Cu_2_O and Bi_12_O_17_Cl_2_ NPs	Nanoparticles in hydrogels	NIR	Hydrogel crosslinking, film formation, and ROS generation	*Staphylococcus aureus*, *Escherichia coli*, and *Streptococcus mutans* [259]	tooth whitening and biofilm removal
Upconversion nanoparticles (UCNPs) and porphyrinic MOFs (PCN-224) NPs doped with L-arginine and incorporated in PVDF electrospun fibers	Nanoparticles in nanofibers	NIR	ROS generation and nitric oxide-assisted photodynamic therapy	*Staphylococcus aureus* and *Pseudomonas aeruginosa* [260]	wound healing
Upconversion nanoparticles (UCNPs) incorporated in PVDF electrospun fibers	Nanoparticles in nanofibers	NIR	ROS generation	*Staphylococcus aureus* and *Escherichia coli* [261]	wound healing
PVA microneedles with a metal-organic framework and multifunctional porphyrin-like metal center NPs	Microneedles	NIR	Photothermal conversion and nanozyme/peroxidase properties of NPs	*Staphylococcus aureus* [260]	wound healing
Iodophilic MOF UiO-66 containing Au nanorods coated with SiO_2_ and embedded in PVP	Nanoparticles in films	NIR	Photoactive nanoparticles	*Staphylococcus aureus* and *Escherichia coli* [262]	nosocomial infections
Ag-sodium lignin sulfonate NPs and polypyrrole-polydopamine NPs in poly(ethylene glycol) diacrylate hydrogel	Nanoparticles in hydrogels	NIR	Photothermal activity and antibacterial Ag ion release	*Staphylococcus aureus* and *Escherichia coli* [263]	wound dressings
PLGA-PCL-methylene blue fibers	Nanofibers	Visible	Controlled matrix degradation and photosensitizer release, photodynamic therapy, and ROS generation	*Escherichia coli* and *Streptococcus mutans* [264]	
Conjugated polymer NPs + cell-penetrating peptides embedded in polyisocyanides hydrogel	Nanoparticles in hydrogels	White and NIR	Synergistic photodynamic and photothermal therapy	*Staphylococcus aureus*, *Escherichia coli*, and *Aspergillus niger* [248]	clinical infections
Light-responsive TiO_2_ nanotubes and thermo-responsive copolymer	Functionalized composite surface	UV	ROS generation	*Staphylococcus aureus* and *Escherichia coli* [265]	anti-adhesion
Porphyrin photosensitizer and PLGA-encapsulated bFGF nanospheres embedded in carboxymethyl chitosan-sodium alginate	Nanoparticles in hydrogels	Visible	Photodynamic chemotherapy	*Staphylococcus aureus* and *MDR-Staphylococcus aureus* [266]	burn wounds
ZnO incorporated with Ag NPs, embedded in carboxymethyl cellulose hydrogel	Nanoparticles in hydrogels	Visible	Ag and Zn ions are released and ROS generation occurs	*Staphylococcus aureus* and *Escherichia coli* [267]	
Porphyrin-based porous organic polymers	Nanoparticles in films	Visible	Photothermal effect and ROS generation	*Methicillin-resistant Staphylococcus aureus* [268]	wound healing
Riboflavin-modified PVC film	Functionalized composite surface	Blue light	ROS generation	*Pseudomonas aeruginosa* [269]	
Hydrogel of polyvinyl alcohol modified with chitosan, polydopamine, and NO release donor/red phosphorous nanofilm	Functionalized composite surface	NIR	Peroxynitrite (ONOO^−^) generation, controlled release, and hyperthermia	*MDR-Staphylococcus aureus* [270]	bone implants
PPy-poly dopamine NPs embedded in NIPAm/acrylic acid hydrogel	Nanoparticles in hydrogels	NIR	Light-triggered tunable hydrogel deformation and adhesion and photothermal therapy	*Staphylococcus aureus* and *Escherichia coli* [271]	wound healing
Ciprofloxacin-loaded PEG hydrogel	Hydrogel	UV	Light-triggered drug release and photo-cleavable molecular cage	*Staphylococcus aureus* [272]	wound healing
Dibenzaldehyde-grafted poly (ethylene glycol), lauric acid-terminated chitosan, and curcumin-loaded mesoporous polydopamine NPs	Nanoparticles in hydrogels	NIR	Light-triggered drug release, hyperthermia with cellular component leakage, and disruption of the bacterial membrane	*Staphylococcus aureus* and *Escherichia coli* [273]	wound healing
Polysaccharide hydrogel encapsulating ferric tannate NPs and vancomycin	Nanoparticles in hydrogels	NIR	Hyperthermia and light-triggered drug release	*Staphylococcus aureus* [274]	wound healing
Prussian blue and tannic acid-loaded polyacrylamide Hydrogel	Hydrogel	NIR	Photothermal therapy	*Staphylococcus aureus* [275]	wound healing
Curcumin-based metal-organic framework + vancomycin, and chitosan	Nanoparticles in hydrogels	NIR	Bacterial capturing, Zn ions, and antibiotic release	*Staphylococcus aureus* [276]	wound healing
TiO_2_ nanorod array	Functionalized composite surface	NIR	Hyperthermia, ROS generation, and bacterial membrane puncture	*Staphylococcus aureus* and *Escherichia coli* [277]	bone implants
Chitosan microspheres loaded with rose bengal and polypyrrole in PVA hydrogel	Nanoparticles in hydrogels	Visible and NIR	Photothermal and photodynamic therapy	*Staphylococcus aureus* and *Escherichia coli* [278]	wound healing
Aloe-Emodin/Carbon Nanoparticle Hybrid PEG hydrogel	Nanoparticles in hydrogels	NIR	ROS generation and drug release	*Staphylococcus aureus* and *Escherichia coli* [279]	wound healing
PVA-(GS-Linker-MPEG) hydrogel loaded with Cy3/Cy5-silica NPs and UCNPs	Nanoparticles in hydrogels	NIR	NIR-UV conversion and light-triggered antibiotic release	*Staphylococcus aureus* [280]	infected wounds
Rose bengal/graphene oxide/PVA/chitosan hybrid hydrogel	Nanoparticles in hydrogels	Visible and NIR	Photothermal therapy and ROS generation	*Staphylococcus aureus* and *Escherichia coli* [281]	wound healing
Photochromic low-MW supramolecular hydrogel, drug loaded	Hydrogel	Visible	Light-triggered hydrogel dissolution and drug release	*Escherichia coli* [282]	
Catechol-conjugated poly(vinylpyrrolidone) sulfobetaine/polyaniline	Polymer coating	NIR	Photothermal therapy	*Staphylococcus aureus* and *Escherichia coli* [283]	
Pectin—Ag/AgCl/ZnO plasmonic hybrid nanocomposites	Nanoparticles in hydrogels	Visible	Photocatalytic nanostructures, ROS generation, and Zn and Ag ion release	*Staphylococcus aureus* and *Escherichia coli* [284]	
Berberine-microalgae/carboxymethyl chitosan/sodium alginate hydrogel	Hydrogel	Visible	Light-triggered drug release, ROS generation, QS downregulation, and inhibition and destruction of the biofilm	*Methicillin-resistant Staphylococcus aureus* [285]	infected wounds
Chlorinated e6-methacrylated silk fibroin	Film	UV and NIR	Photodynamic therapy	*Staphylococcus aureus* [286]	surgical wounds
Dopamine-folic acid hydrogel loaded with transition metal ions + carbon quantum dot-decorated ZnO NPs	Nanoparticles in hydrogels	Visible and NIR	Photothermal therapy, ROS generation, Zn ion release, and bacteria wall penetration	*Staphylococcus aureus* and *Escherichia coli* [287]	wound healing

Another interesting mechanism of biofilm disruption is based upon the photo-induced release of nitric oxide (NO) and its derivatives in the EPS microenvironment [239,246,255,289]. Self-assembled micelles were formed with a diblock copolymer of poly-ethylene glycol and N-nitrosamine fragments, functionalized with a coumarin chromophore (PEO-b-PCouNO), and loaded with Ciprofloxacin [246]. The micelles were synthesized to respond to a visible light stimulus and simultaneously release the antibiotic compound and NO molecules to synergistically disperse *Pseudomonas aeruginosa* biofilm and kill the bacterial cells. The NO-antibacterial action is known to be multiple (Figure 7): (i) NO^●^ can move across the bacterial membrane and initiate extra- and intracellular nitrosative stress cascades with the generation of oxidative by-products, lipid peroxidation, and nitration of membrane-bound proteins, which ultimately lead to RNA and DNA damage; (ii) NO can interfere with the quorum sensing pathway at a molecular level via nitrosylation of transcriptional regulators and enzymes (i.e., LasR, RhIR, MvfR, PqsD, and PqsE) [289] and, consequently, disperse bacterial biofilm.

### 6.2. Light-Responsive Hydrogels and Polymeric Composite Structures

Polymeric and hydrogel-based composite structures are promising antibacterial systems in applications such as wound healing, surgical sutures, tissue engineering, and dental procedures where good compliance is needed at the material and the tissue/organ interface. The light responsiveness of macromolecule-based materials may be due either to the presence of light-responsive moieties within the polymeric chain or to the integration of photoactivatable agents in the polymeric matrix. Table 3 reports a representative list of the most recent literature on the subject.

Hydrogels are defined as three-dimensionally cross-linked macromolecular networks able to swell as a result of intense water absorption. Depending on their mechanism of action, hydrogels may present intrinsic antibacterial properties (i.e., if formulated with antimicrobial peptides or with cationic polymers such as chitosan), or they can acquire a bactericidal effect when loaded with antibiotics, metal nanoparticles, or metal-organic frameworks (MOFs) [25,29,259,262,276]. The combination of active agents (either organic or inorganic) with a polymer- or hydrogel-like matrix appears to be the most interesting route, as it provides a tailorable platform with multi-functionality potential. Photo-activated antibacterial hydrogels exert their function upon irradiation under a specific wavelength that excites a photosensitizer (or a chromophore compound) embedded in the 3D network, which in turn triggers an oxidation cascade in the materials surrounding ROS generation (Figure 7) [29]. In parallel, the hydrogel matrix may undergo a photo-induced change in its mechanical properties and degrade or dissolve, thus slowly leaching out additional antibiotic cargos that perpetrate the antimicrobial action [271,272].

Qiao and colleagues [280] proposed a smart hydrogel with the dual purpose of monitoring the bacterial infection in wounds by means of a fluorescent light and releasing an antibiotic compound on demand upon NIR stimulation. The matrix of the hydrogel was constituted of polyvinyl alcohol (PVA) and an UV-cleavable polyprodrug (GS-Linker-MPEG); within this 3D network, multifunctionalities were included: (i) Cy3 and Cy5-modified silica nanoparticles (SNP-Cy3/Cy5) as sensing agents for bacterial infection; and (ii) up-conversion nanoparticles (UCNP) responsible for the prodrug UV-cleavage. Upon NIR irradiation, UCNPs were able to convert NIR light into UV light to activate the release of the GS drug from the polymeric network. In a different study, Feng et al. [271] developed a N-isopropylacrylamide and acrylic acid hydrogel that was able to tune its adhesion and deformation when subjected to NIR illumination. The hydrogel contained conductive polypyrrole and polydopamine PPy-PDA nanoparticles that can transfer NIR light energy into heat, reaching temperatures up to 58.7 °C within 10 min of stimulation and demonstrating photothermal converting ability. Moreover, as a consequence of the increased temperature in the material, water molecules rearrange within the 3D network while inter-polymer chain associations and hydrophobic interactions take place, thus causing an asymmetric shrinkage of the hydrogel and its self-deformation.

As a different mechanism of action, Hu and co-workers [285] targeted the biofilm QS of Methicillin-resistant *Staphylococcus aureus* (MRSA) with a composite hydrogel of microalgae *Spirulina platensis* and carboxymethyl chitosan/sodium alginate, loaded with berberine, a bioactive molecule known for its antibacterial effect and its ability to slow down some of the QS-associated processes. The berberine-loaded hydrogel promoted ROS formation in the wound microenvironment while suppressing biofilm formation and, more interestingly, down-regulating the expression of multi-resistant virulence.

The NIR-induced antimicrobial photothermal effect has also been widely documented in the literature for hydrogel-based systems, either alone or in combination with the photodynamic effect [231,275,283,287].

Table 3 reports other examples of light-triggered composite materials, such as microneedles [260], electrospun fibers [260,264], and functional coatings [270,283]. The range of shapes and 3D arrangements easily obtained when working with polymeric substrates enables the realization of multi-functional antibacterial strategies with spatial and temporal control of the photoactivation. Future developments must converge into hybrid approaches based on the combination of an organic matrix and inorganic compounds, which can benefit from the use of a broad spectrum of wavelengths to tackle the growth of microorganisms and the formation of biofilm.

## 7. Conclusions

The use of light to fight bacterial spreading and antimicrobial resistance can provide several advantages, such as: (i) the targeted action conferred by the light stimulus; (ii) the controlled release of drug action (both from photoswitchable molecules or light-responsive materials) in terms of dosing and timing without the risk of overusing antimicrobials or the risk of systemic effects associated with traditional antibiotic therapies; and (iii) a reduced risk of antimicrobial resistance occurrence since the photoswitchable drugs can be spread in their inactive form with a reduced environmental impact. Finally, light-triggered approaches can be coupled with other therapeutic strategies (i.e., conventional antibiotics) to provide a combination therapy approach to tackle the complex problem of bacterial infections and biofilm spreading.

However, there are also several challenges associated with the use of light-activated compounds for antimicrobial applications. One of the main challenges relates to the optimal delivery of compounds to the biofilm [290]. Biofilms are complex and heterogeneous structures, and the matrix that surrounds the bacteria can limit the penetration of compounds. To this end, the use of nanoparticles or other delivery systems can be of great help. Another challenge lies in the optimization of the activation process. UV light has a cytotoxic effect and should be avoided as a trigger for photosensitive compounds. Therefore, researchers are working to provide switchable compounds and materials that can be addressed with less harmful light triggers (i.e., near-infrared NIR light). Toxicity of light towards cells may happen in photothermal therapy, where the NIR light stimulation leads to an increase in the temperature of the material and, consequently, of its surroundings, causing necrotic effects within bacterial cells. When in contact with human/patient tissue, this can be a potential issue, which could be limited by developing strategies to localize the NIR illumination onto the light-responsive material or by decorating the light-responsive materials with targeting moieties for biofilm adhesion and penetration. Moreover, the light trigger needs to be tuned according to the chemical structure of the light-sensitive system, and the intensity and duration of light exposure may also need to be carefully tuned. Hence, it is important to develop methods for precisely controlling the activation of the compounds within the biofilm. Furthermore, the development of resistance to light-activated compounds is a potential concern. Bacteria may evolve mechanisms to avoid or resist the effects of such compounds, and it is therefore important to understand and address these mechanisms to ensure the long-term effectiveness of photopharmacological approaches. Finally, there are regulatory and safety considerations that need to be addressed when developing photopharmacological strategies for bacterial biofilm control. The safety of light-activated compounds and their potential impact on non-target organisms and the environment must be carefully evaluated.

In summary, the challenges of photopharmacology for bacterial biofilm control include optimizing the delivery and activation of the light-sensitive compounds, addressing the potential for resistance, and ensuring that regulatory and safety considerations are met.

As of today, the main drawbacks of light-triggered materials are related to their potential cytotoxicity, hemolysis, metabolic toxicity, and difficult body/tissue clearance when administered, for example, to human patients. Such safety issues may limit their applications in the biomedical field and should be overcome in order to implement nanotherapeutic strategies to efficiently battle microbial infections and bacterial resistance in clinical settings [26,236].

## Figures and Tables

**Table 1:** Biofilm disruption strategies classified by mechanism of action.

*Mechanism of Action and Target Mechanism*		*Approach/Molecule with Antibiofilm Activity*	*Resulting Phenotype*	*Target Bacteria*
***Targeting adhesion strategy of bacteria***	Genetic engineering	Deletion/mutation of the *UpaB* gene	Blocks the super adhesion protein (UpaB) to Inhibit biofilm formation	*Escherichia coli* (UTI infections) [108]
	Enzyme activity inhibition	Myricetin (a flavonoid)	Inhibits Sortase A, an enzyme that catalyzes initial adhesion between *Streptococcus mutans* surface protein Pac and lectin, thus prohibiting surface adhesion	*Streptococcus mutans* [109]
	Deactivation of adhesive protein	D-arabinose	Prevents the adhesion of oral bacteria to dental implant surfaces through lectin	*Streptococcus oralis*, *Fusobacterium nucleatum,* and *Porphyromonas gingivalis* [110]
	Peptide mimic of bacterial α-helical peptide	Designed helical peptide G(IIKK)3I-NH2 (G3)	Prevents biofilm formation by acting on bacterial surface adhesion parameters; degrades eDNA to destabilize the mature biofilm architecture	*Streptococcus mutans* in tooth infections [111]
	Competition for surface binding sites	Nanoparticles coated with the outer membrane of *Helicobacter pylori*	Compete with bacteria for binding sites on host gastric epithelial cells and inhibit bacterial adhesion	*Helicobacter pylori* [112]
	Inhibiting Multivalent Adhesion Molecules (MAMs)	MAM7-mimicking inhibitor	Competes with the pathogen for sites on the host to initiate an infection	*Mutidrug resistant* and *Pseudomonas aeruginosa* in burn wounds [113]
***Surface antiadhesion***	Medical devices with antiadhesive and antimicrobial properties	Grafting 2-methacryloyloxyethyl phosphate choline (MPC) onto medical devices	Prevents nonspecific adsorption of proteins and bacteria, resulting in biofilm inhibition	Gram-negative and Gram-positive bacteria [114]
		Application of a surface coating called mPep (mixed-charge polypeptide) in medical catheters in vivo	Reduces bacterial adhesion	Methicillin-resistant *Staphylococcus aureus* and *Pseudomonas aeruginosa* [115]
		Polydopamine-coated membranes with integerated copper nanoparticles (CuNPs)	Decrease *Escherichia coli* viability by 76%	*Escherichia coli* [116]
		Modifying the polypropylene surface by reactive ion-etching technology	Reduce *Escherichia coli* adhesion on pp surfaces	*Escherichia coli* [117]
***Targeting Biofilm formation strategy***	Destruction of EPS	Deoxyribonuclease I (DNase I)	Degradation of eDNA, resulting in the prevention of adhesion	*Staphylococcus aureus* and *Pseudomonas aeruginosa* [118]
		Alginate lyase	Alginate degradation results in the prevention of biofilm formation	*Pseudomonas aeruginosa* [119]
		Proteases	Hydrolysis of matrix proteins and adhesins in EPS	*Staphylococcus aureus* [120]
		Cellulase	Degrades EPS	*Burkholderia cepacia* [121]
		α-Amylase	Mature biofilm degradation after EPS disruption	*Staphylococcus aureus* [122]
	Inhibition of QS signal molecule production	FabI derivatives	Inhibition of enoyl-ACP reductase, which contributes acyl chain lengths of N-acyl homoserine lactones	*Pseudomonas aeruginosa* [123]
		(2-nitrophenyl) methanol derivatives	Inhibitors of PqsD, a key enzyme of signal molecule biosynthesis	*Pseudomonas aeruginosa* [124]
		TNRHNPHHLHHV (peptide)	Inhibits LuxS enzyme activity to Inhibit AI-2 production	*Streptococcus suis serotype 2 (SS2)* [125]
	Degradation of the QS Signaling molecule	AHL-lactonase AiiA	Degrades AHLs to prevent biofilm formation	*Pseudomonas aeruginosa*, *Vibrio cholerae*, and *Enterobacter cloacae* [126]
		Boronic acid derivate SM23	Decreases the signaling molecules 3-oxo-C12-HSL and C4-HSL to reduce biofilm formation	*Pseudomonas aeruginosa* [127]
		3-(dibromomethylene) isobenzofuran-1(3H)-one derivatives	Inhibits biofilm formation through the inhibition of AI-2 activity	*Fusobacterium nucleatum*, *Porphyromonas gingivalis*, and *Tannerella forsythia* [128]
		Diketopiperazines	Interferes with the activity of signal molecule synthase CepI, resulting in the prevention of biofilm formation	*Burkholderia cenocepacia* [129]
		L-carvone	Inhibits QS activity by reducing AHL production (0.5 µL/mL)	*Hafnia alvei* [130]
		Acylase	Cleaves the amide bond of AHLs	Gram-negative bacteria [131]
		Imidazole	Degrades AI-2	*Escherichia coli* [132]
		Epigallocatechin gallate	Interferes with AHL production and AI-2-mediated QS	*Staphylococcus aureus*, *Burkholderia cepacia*, and *Eikenella corrodens* [133,134,135]
		Acyl-HSL analog J8-C8	Disturbs QS molecule C8-HSL synthesis and affects biofilm formation	*Burkholderia glumae* [136]
	Targeting QS Signaling Receptors	3-Phenyllactic acid (PLA)	Binds with QS receptors RhlR and PqsR involved in biofilm formation	*Pseudomonas aeruginosa* [137]
		Furanones and synthetic furanones	Competes with the native autoinducers to bind to the AHL receptors, decreasing virulence factor production and biofilm formation	*Pseudomonas aeruginosa* [138,139]
		Sesquiterpene lactone	Decreases the affinity of the CviR protein to its receptor, LuxR	*Chromobacterium violaceum* [140]
		Naringenin, Taxifolin, and Quercetin 4′-O-β-Dglucopyranoside	Inhibit QS-regulated gene expression. Reduce QS via the vfr-mediated lasIR system	*Pseudomonas aeruginosa* [141]
		N-(3-oxododecanoyl) homoserine lactone derivatives	Block the binding site of the QS molecule, inhibiting biofilm formation and increasing antibiotic sensitivity	*Pseudomonas aeruginosa* clinical strains [142]
		Flavonoids compounds	Reduce QS signal concentration	*Yersinia enterocolitica* [143]
		A small peptide 5906	Prevents homodimer formation, inhibiting LuxS activity	*Edwardsiella tarda* [144]
		D-galactose	Inhibit AI-2 activity	Periodontopathogens [145]
		N-phenyl-4-(3-phenylthioureido) benzenesulfonamide	Allosterically modifies the AI-3 receptor QseC, impedes virulence expression, and promotes biofilm formation	*Escherichia coli (EAEC) O104:H4* [146]
	Blocking the QS Signaling Cascade	Savirin	Targets *AgrA* to disrupt *Agr* operon-mediated QS	*Staphylococcus aureus* [73]
		Curcumin	Inhibits QS-controlled protease and biofilm formation	*Pseudomonas aeruginosa PAO1* [147]
		Efflux pump inhibitor PAβN	Reduces the extracellular accumulation of QS signals and diminishes the relative expression of the QS cascade (pqsA, pqsR, lasI, lasR, rhlI, and rhlR)	*Pseudomonas aeruginosa* clinical isolate [148]
***Targeting mature biofilm***	Killing persister and dormant cells	Adenosine (ADO)	Activates ATP and GTP synthesis and promotes cell respiration, thereby enhancing the killing of persistent cells by antibiotics	Gram-negative and Gram-positive bacteria [149]
		Dialylquinoline TMC207	Targets ATP synthase, thereby damaging the lipopeptide of the bacterial membrane, including persistent cells, and effectively improving the antibiotic treatment success rate	*Mycobacterium tuberculosis* [150]
		Lead compound X9	Inhibition of the RelMtb enzyme that is used to enter the persister cell stage. Inhibition of this enzyme kills the persister cells directly	*Mycobacterium tuberculosis* [151]

**Table 2:** Light-triggered anti-bacterial nano-formulations.

Materials	Formulation	Light	Mechanism	Target Microorganism	Application
ZnO-based and squaraine nanohybrids	Nanoparticles	NIR	NP internalization, photoinduced interfacial electron transfer, and ROS generation to disrupt bacterial biofilm	*Staphylococcus aureus* [240]	artificial implants
Au core in shell-based mesoporous silica nanoparticles	Nanoparticles	NIR	Incorporation of nitrosothiol groups (-SNO) with a heat-liable linker, NO release upon photothermal stimulation, and antimicrobial Levofloxacin to disrupt bacterial biofilm	*Staphylococcus aureus* [239]	
PEG-b-pLAMA/pAAPBA-b-pDPA NPs loaded with indocyanine green	Nanoparticles	NIR	Acidic infection environment, borate ester linkage cleavage, NP shelling, hyperpyrexia, and ROS generation	*Pseudomonas aeruginosa* [243]	clinical chronic infections in pulmonary alveoli
Distearoyl phosphatidylcholine + betainylate cholesterol micelles loaded with Cypate and Tobramycin	Liposomes	NIR	Photothermal and antibiotic synergy, penetration into biofilm channels, and thermal-triggered drug release	*Pseudomonas aeruginosa* [233]	
Core-shell upconversion nanoparticles and TiO_2_(UCNPs@TiO_2_)	Nanoparticles	NIR and UV	Under NIR light, the core UCNPs emit UV light, which triggers the photodynamic function of the shell via energy transfer	*Streptococcus sanguinis*, *Porphyromonas gingivalis*, and *Fusobacterium nucleatum* [241]	periodontitis
Graphene oxide—Hyaluronic acid—Ag NPs	Nanoparticles	NIR	GO absorbs light and generates heat; hyaluronidase-triggered photothermal platform and ROS generation	*Staphylococcus aureus* [244]	
2-nitrobenzaldehyde-modified zeolitic imidazolate mesoporous NPs + rifampicin	Nanoparticles	UV	UV-light (365 nm)-responsive in situ production of acid, pH-dependent degradation of the zeolite framework, antibiotic release	*Staphylococcus aureus* and *Methicillin-resistant Staphylococcus aureus* [245]	wound healing
Nitric oxide-releasing micelles, PEO-b-polyCouNO + Ciprofloxacin	Nanomicelles	Visible	visible light irradiation, fluorescence turn-on, enabling in situ self-reporting NO release fluorescence turn-on (>185-fold)	*Pseudomonas aeruginosa* [246]	
Ag_3_PO_4_ NPs loaded on Bi_2_S_3_ nanorods	Nanorods	NIR	ROS generation after enhanced photocatalytic effect via photoactive nanoparticles and semiconductor heterojunction coating	*Staphylococcus aureus* and *Escherichia coli* [247]	orthopedic implants
Ag-Cu_2_O/PANI	Nanoparticles	Visible	Intracellular ROS generation is enhanced by photocatalytic particles and conductive polymer	*Staphylococcus aureus* and *Pseudomonas aeruginosa* [248]	
Black phosphorus quantum dots + vancomycin in a liposome	Liposomes	NIR	Hyperthermia, light-trigger liposome disruption, and release of its antibiotic cargo	Methicillin-resistant *Staphylococcus aureus* [234]	skin infections
Mesoporous polydopamine NPs with the photosensitizer Indocyanine Green	Nanoparticles	NIR	Photothermal/photodynamic therapy	*Staphylococcus aureus* [249]	bone implants
MoSe_2_/TiO_2_ Nanorod	Nanorods	NIR	Photothermal/photodynamic therapy	*Staphylococcus aureus* and *Escherichia coli* [250]	bone implants
2,2′-(ethylenedioxy)bis (ethylamine) functionalized Carbon Nanodots	Nanodots	Visible	Breakage of the EPS matrix	*Bacillus subtilis* [235]	
Halogen/nitrogen-doped polymeric graphene quantum dots	Quantum dots	LED	ROS generation	*Staphylococcus epidermidis*, *Staphylococcus aureus*, and *Pseudomonas aeruginosa* [251]	wound healing
CuS nanoparticles + photoacid generator	Nanoparticles	Visible	Intramolecular photoreaction, pH decrease, and peroxidase activity	*Staphylococcus aureus* and *Escherichia coli* [252]	
Chitosan-coated silver NPs and graphene nanoribbon nanowires	Nanoparticles	NIR	Photothermal therapy	*Pseudomonas aeruginosa* [253]	medical patches
Graphene quantum dots + erytromycin + mesoporous silica NPs	Nanoparticles	LED	Singlet oxygen production, photodynamic therapy, and drug release	*Staphylococcus aureus* and *Escherichia coli* [238]	wound healing
Core-shell ZnFe_2_O_4_/AgCl@EDTA-Ag composites	Nanoparticles	Visible	Photocatalytic nanostructures and ROS generation	*Escherichia coli* [254]	medical disinfection
Poly(selenoviologen)-Assembled Upconversion Nanoparticles	Nanoparticles	NIR	Photothermal and photodynamic therapy and ROS generation	Methicillin-resistant *Staphylococcus aureus* [242]	infected wounds
Carbon nanodots + curcumin	Nanodots	Visible and NIR	Photothermal and photodynamic therapy and ROS generation	*Staphylococcus aureus* and *Escherichia coli* [236]	
Au plasmonic NPs	Nanoparticles	Visible	Nanostructure clusterization and photothermal effect	*Escherichia coli* [237]	
Polydopamine-Fe_2_O_3_ NPs with NONOates and dendritic poly(amidoamine)	Nanoparticles	NIR	Photothermal therapy and controlled NO release	*Staphylococcus aureus* and *Escherichia coli* [255]	

## Data Availability

Data sharing not applicable.
